# Origins of *De Novo* Genes in Human and Chimpanzee

**DOI:** 10.1371/journal.pgen.1005721

**Published:** 2015-12-31

**Authors:** Jorge Ruiz-Orera, Jessica Hernandez-Rodriguez, Cristina Chiva, Eduard Sabidó, Ivanela Kondova, Ronald Bontrop, Tomàs Marqués-Bonet, M.Mar Albà

**Affiliations:** 1 Evolutionary Genomics Group, Hospital del Mar Research Institute (IMIM), Barcelona, Spain; 2 Department of Experimental and Health Sciences, Universitat Pompeu Fabra (UPF), Barcelona, Spain; 3 Proteomics Unit, Universitat Pompeu Fabra (UPF), Barcelona, Spain; 4 Proteomics Unit, Centre de Regulació Genòmica (CRG), Barcelona, Spain; 5 Biomedical Primate Research Center (BPRC), Rijswijk, The Netherlands; 6 Centro Nacional de Análisis Genómico (CNAG), Barcelona, Spain; 7 Institució Catalana de Recerca i Estudis Avançats (ICREA), Barcelona, Spain; Yale University, UNITED STATES

## Abstract

The birth of new genes is an important motor of evolutionary innovation. Whereas many new genes arise by gene duplication, others originate at genomic regions that did not contain any genes or gene copies. Some of these newly expressed genes may acquire coding or non-coding functions and be preserved by natural selection. However, it is yet unclear which is the prevalence and underlying mechanisms of *de novo* gene emergence. In order to obtain a comprehensive view of this process, we have performed in-depth sequencing of the transcriptomes of four mammalian species—human, chimpanzee, macaque, and mouse—and subsequently compared the assembled transcripts and the corresponding syntenic genomic regions. This has resulted in the identification of over five thousand new multiexonic transcriptional events in human and/or chimpanzee that are not observed in the rest of species. Using comparative genomics, we show that the expression of these transcripts is associated with the gain of regulatory motifs upstream of the transcription start site (TSS) and of U1 snRNP sites downstream of the TSS. In general, these transcripts show little evidence of purifying selection, suggesting that many of them are not functional. However, we find signatures of selection in a subset of *de novo* genes which have evidence of protein translation. Taken together, the data support a model in which frequently-occurring new transcriptional events in the genome provide the raw material for the evolution of new proteins.

## Author Summary

For the past 20 years scientists have puzzled over a strange-yet-ubiquitous genomic phenomenon; in every genome there are sets of genes which are unique to that particular species i.e. lacking homologues in any other species. How have these genes originated? The advent of massively parallel RNA sequencing (RNA-Seq) has provided new clues to this question, with the discovery of an unexpectedly high number of transcripts that do not correspond to typical protein-coding genes, and which could serve as a substrate for this process. Here we have examined RNA-Seq data from 8 mammalian species in order to define a set of putative newly-born genes in human and chimpanzee and investigate what drives their expression. This is the largest-scale project to date that tries to address this scientific question. We have found thousands of transcripts that are human and/or chimpanzee-specific and which are likely to have originated *de novo* from previously non-transcribed regions of the genome. We have observed an enrichment in transcription factor binding sites in the promoter regions of these genes when compared to other species; this is consistent with the idea that the gain of new regulatory motifs results in *de novo* gene expression. We also show that some of the genes encode new functional proteins expressed in brain or testis, which may have contributed to phenotypic novelties in human evolution.

## Introduction

New genes continuously arise in genomes. Recent evolutionary 'inventions' include small proteins that have functions related to the adaptation to the environment, such as antimicrobial peptides or antifreeze proteins, which have independently evolved in different groups of organisms [1,2]. A well-studied process for the formation of new genes is gene duplication and subsequent sequence divergence [3,4]. However, in recent years another important mechanism for the birth of new functional genes has been discovered- *de novo* gene emergence [5–7]. As deduced by comparisons to the genomic syntenic regions in other species, these genes derive from previously non-genic regions of the genome [8–14]. Genes that have recently evolved *de novo* are characterized by their lack of homologous genes in other species and, contrary to duplicated genes, they can evolve without the limitations which constrain sequences that have high similarity to a pre-existing gene [15]. Despite their recent origin, it has been shown that *de novo Drosophila* genes can quickly become functionally important [13,16].

Species or lineage-specific genes, which are often called orphan genes, have been described in a wide range of organisms, including yeast [9,17,18], primates [12,19–21], rodents [10,11,22], insects [8,23–25], and plants [26,27]. These studies based on annotated protein-coding genes have revealed that orphan genes tend to have a simple gene structure, a short protein size, and are preferentially expressed in one tissue [28,29]. As orphans lack homologues in other species, many of these genes are likely to have arisen *de novo*. Some of these proteins have been functionally characterized. One example is the hominoid-specific antisense gene, NCYM, which is over-expressed in neuroblastoma; this gene inhibits the activity of glycogen synthase kinase 3β (GSK3β), which targets NMYC for degradation [30].

Massively parallel RNA sequencing (RNA-Seq) has revealed that a large fraction of the genome extending far beyond the set of annotated genes is transcribed [31,32] and possibly translated [33–37]. Many genes that are annotated as long non-coding RNAs (lncRNAs) are lineage-specific and display high transcriptional turnover [38,39]. The high transcriptional activity of the genome provides abundant raw material for the birth of new genes. Indeed, the use of transcriptomics data has led to the discovery of an unexpectedly high number of recently emerged genes in yeast [33] and *Drosophila* [40,41]. As most of these genes show little evidence of selection, they have been called 'protogenes' [33]. The products resulting from the expression of protogenes become exposed to natural selection. If useful, they will be retained and continue to evolve under selective constraints [29,42,43].

Here we use transcriptomics data from four mammalian species to quantify the amount of transcription that is human and/or chimpanzee-specific and investigate the molecular mechanisms driving the expression of these transcripts. The data is used to assemble transcripts and identify both annotated and novel genes. The majority of *de novo* genes originate from regions with conserved genomic synteny in macaque. Analysis of these regions reveals that the expression of the genes is associated with the gain of novel regulatory motifs in the promoter region and U1snRNP splice sites downstream of the transcription start site. We also show that at least a subset of the newly evolved genes is likely to encode functional proteins.

## Results

### Assembly of annotated and novel transcripts from strand-specific RNA-Seq data

We used strand-specific sequencing of polyadenylated RNA (polyA+ RNA-Seq) from several tissues from human, chimpanzee, macaque, and mouse, to perform transcript assembly with Cufflinks [44]. The total number of RNA-Seq datasets was 43, of which 26 were generated in this study and the rest were public datasets from previous studies [20,38,45]. The set of tissues sampled included testis and brain; these tissues have been found to be enriched in *de novo* genes [20,46]. In this study, we will use the term 'gene' to refer to the set of transcripts merged into a single locus by Cufflinks. Any genome unmapped reads were assembled *de novo* with Trinity for the sake of completeness [47].

**Fig 1:**
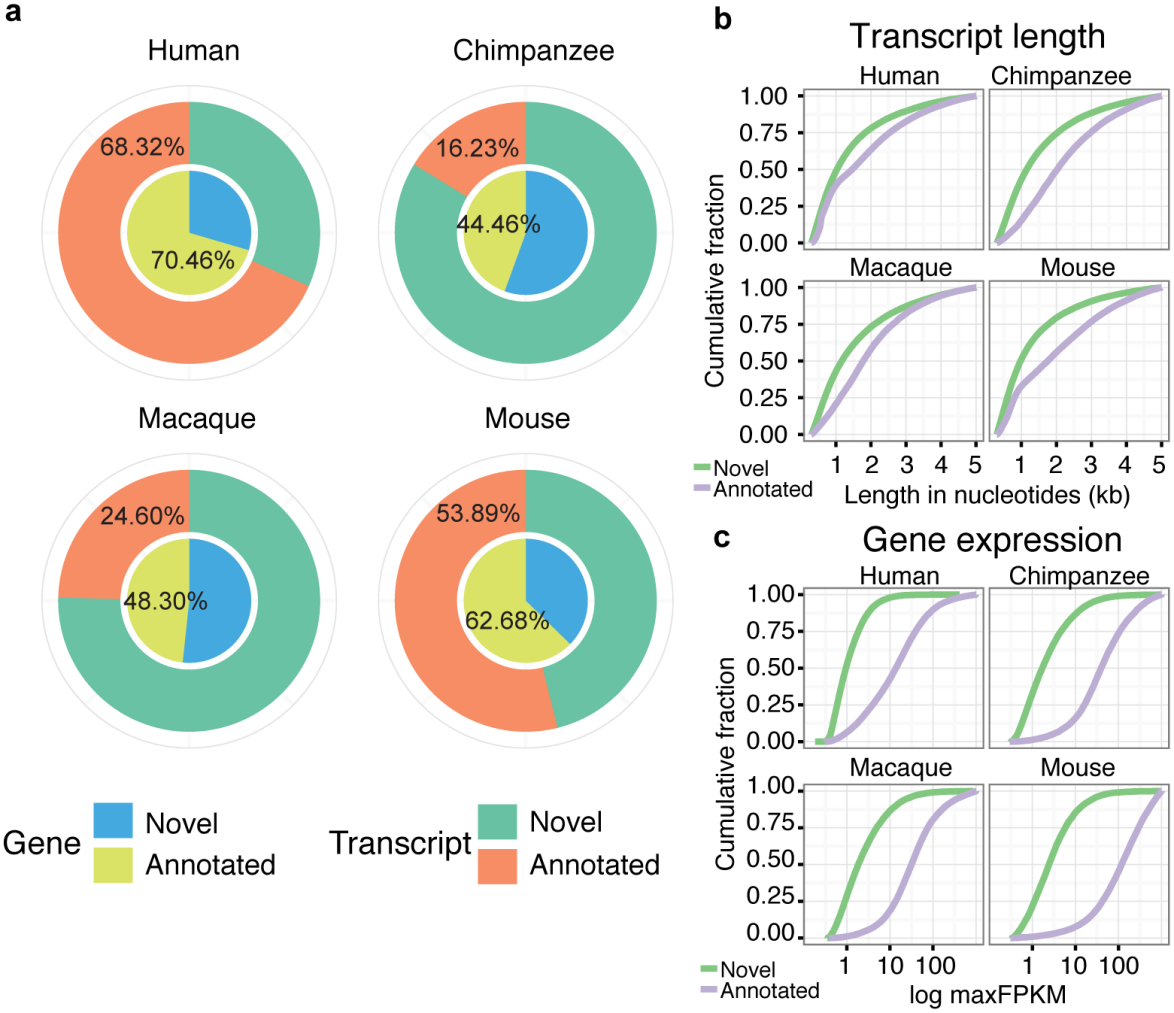
Global properties of assembled transcriptomes. **a)** Percentage of annotated and novel genes and transcripts using strand-specific deep polyA+ RNA sequencing. Classification is based on the comparison to reference gene annotations in Ensembl v.75. 70.65 and 87.77% of annotated genes in human and mouse are classified as protein-coding, respectively. Number of genes identified: human 34,188; chimpanzee, 35,915; macaque 34,427; mouse 31,043. Number of transcripts identified: human 99,670; chimpanzee 102,262; macaque 93,860; mouse 85,688. **b)** Cumulative density of nucleotide length in annotated and novel assembled transcripts. **c)** Cumulative density of expression values in logarithmic scale in annotated and novel assembled transcripts. Expression is measured in fragments per kilobase per million mapped reads (FPKM) values, selecting the maximum value across all samples.

Subsequently, we selected transcripts longer than 300 nucleotides (nt). This excluded any sequencing artifacts resulting from one single amplified paired end read (2x100 nt). We also filtered out all genes with a per-base read coverage lower than 5 to ensure transcript completeness (see Materials and Methods). A negative control lacking reverse transcriptase in the library construction step (RT-) indicated that the probability of a transcript to have resulted from DNA contamination was very low, virtually 0 in the case of multiexonic transcripts. To ensure a highly robust set of transcripts we filtered out intronless genes. This also removed possible promoter- or enhancer associated transcripts (PROMPTS and eRNAs). As a result of this process, we recovered 99,670 human, 102,262 chimpanzee, 93,860 macaque and 85,688 mouse transcripts merged in 34,188 human, 35,915 chimpanzee, 34,427 macaque, and 31,043 mouse gene loci. This included a large fraction of the long multiexonic genes annotated in Ensembl plus a significant number of non-annotated genes (Fig 1a). The number of annotated genes was much higher in human and mouse than in chimpanzee and macaque, mostly due to differences in the number of annotated lncRNAs. About 48% of the human genes not annotated by Ensembl matched genes assembled in recent large-scale RNA-Seq studies [38,48] (S1 Fig). Unsurprisingly, novel genes were shorter and expressed at lower levels than annotated genes (Fig 1b and 1c, respectively). In humans, unannotated genes represented 0.5–2% of the transcriptional cost depending on the tissue, as measured in terms of sequencing reads.

### Identification of *de novo* genes in human and chimpanzee

**Fig 2:**
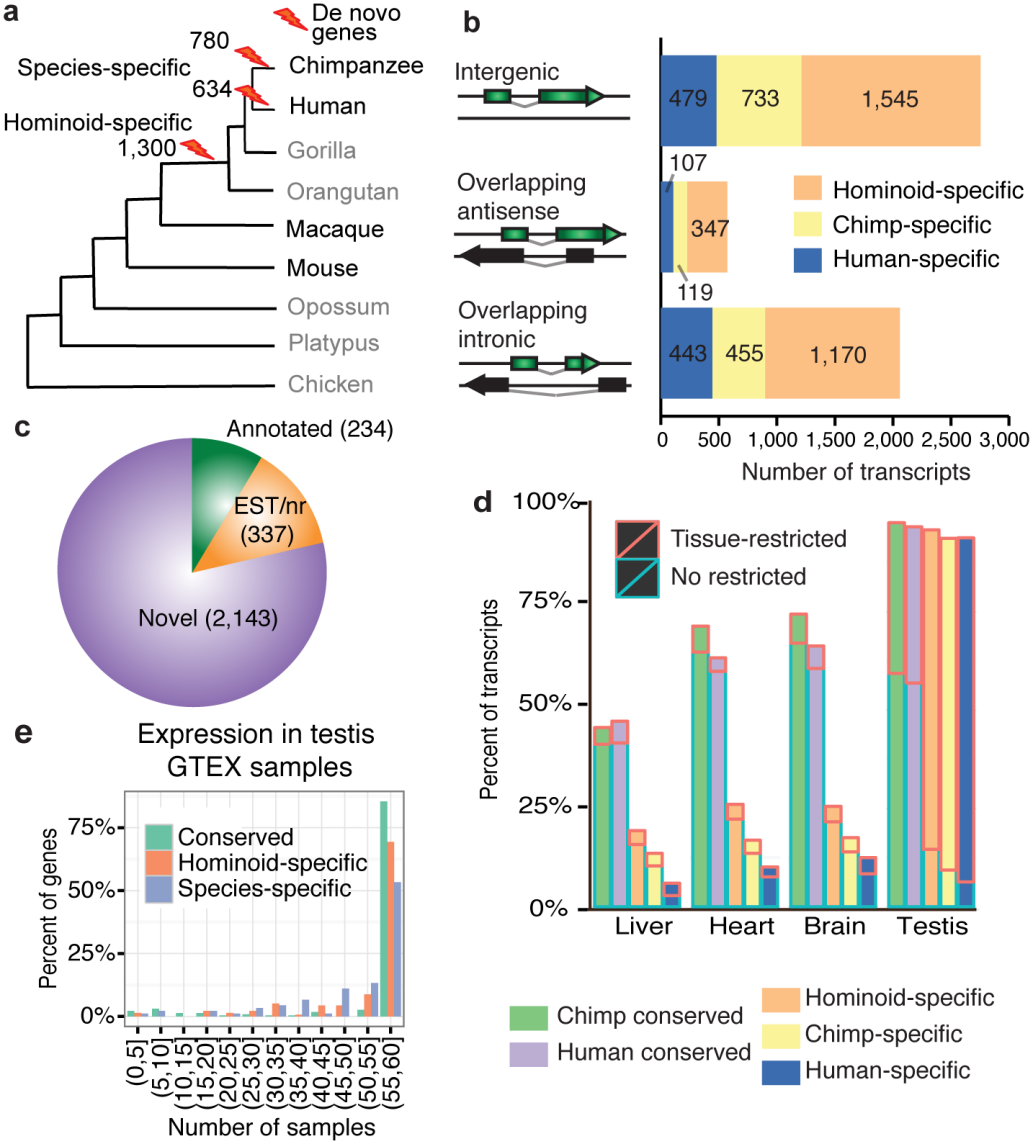
Identification and characterization of *de novo* genes in human and chimpanzee. **a)** Simplified phylogenetic tree indicating the nine species considered in this study. In all species we had RNA-Seq data from several tissues. Chimpanzee, human, macaque and mouse were the species for which we performed strand-specific deep polyA+ RNA sequencing. We indicate the branches in which *de novo* genes were defined, together with the number of genes. **b)** Categories of transcripts in *de novo* genes based on genomic location. Intergenic, transcripts that do not overlap any other gene; Overlapping antisense, transcripts that overlap exons from other genes in the opposite strand; Overlapping intronic, transcripts that overlap introns from other genes in the opposite strand, with no exonic overlap. **c)** Classification of *de novo* genes based on existing evidence in databases. Annotated; genes classified as annotated in Ensembl v.75; EST/nr; non-annotated genes with BLAST hits (10^−4^) to expressed sequence tags (EST) and/or non-redundant protein (nr) sequences in the same species. Novel; rest of genes. **d)** Patterns of gene expression in four tissues. Brain refers to frontal cortex. Transcripts with FPKM > 0 in a tissue are considered as expressed in that tissue. In red boxes, fraction of transcripts whose expression is restricted to that tissue (τ > 0.85, see Methods). Chimp conserved, transcripts assembled in chimpanzee not classified as *de novo*. Human conserved, transcripts assembled in human not classified as *de novo*. **e)** Number of testis GTEx samples with expression of *de novo* and conserved genes. We considered all annotated genes with FPKM > 0 in at least one testis sample. Conserved, genes sampled from the total pool of annotated genes analyzed in GTEx with the same distribution of FPKM values than in annotated *de novo* genes (n = 200).

Next, we used BLAST-based sequence similarity searches [49] to identify the subset of *de novo* genes that could have originated in human, chimpanzee, or the common ancestor of these two species since the divergence from macaque (hominoid-specific genes). These genes lacked homologues in other species after exhaustive searches against the transcript assemblies described above, the transcript assemblies obtained using previously published non-stranded single read RNA-Seq data for nine vertebrate species [50], Ensembl gene annotations for the same set of species, and the complete expressed sequence tag (EST) and non-redundant (nr) protein databases from NCBI. We also employed genomic alignments to discard any transcripts expressed in syntenic regions in other species that could have been missed by BLAST (S2 Fig). This pipeline identified 634 human-specific genes (1,029 transcripts), 780 chimpanzee-specific genes (1,307 transcripts), and 1,300 hominoid-specific genes (3,062 transcripts). Taken together, the total number of candidate *de novo* genes was 2,714 (5,398 transcripts) (Fig 2a). The rest of genes will be referred to as conserved genes.

As we used strand-specific RNA sequencing, we could unambiguously identify a large number of antisense transcripts. Many of them were located within intronic regions (38.31%) and others partially overlapped exonic regions of other genes (10.62%). The rest of *de novo* transcripts were located in intergenic regions (51.07%). These percentages were similar for human, chimpanzee, and hominoid-specific genes (Fig 2b). Eight *de novo* genes from human and/or chimpanzee matched annotated protein-coding genes (S1 Table). One example was *GTSCR1* (Gilles de la Tourette syndrome chromosome region, candidate 1), encoding a 137 amino acid long protein with proteomics evidence. Curiously, the human protein-coding genes in this set, including GTSCR1, were annotated as long non-coding RNAs (lncRNAs) in a subsequent Ensembl version (77). About 20% of *de novo* genes matched annotated lncRNAs or sequence entries in the 'EST' or 'nr' databases (Fig 2c). *De novo* transcripts had a similar distribution along the chromosomes than the rest of assembled transcripts (S3 Fig).

Transcripts from *de novo* genes were shorter and expressed at lower levels than those from conserved genes (S4 Fig). These biases have also been noted in young annotated primate protein-coding genes [12,20]. In general, *de novo* genes were located in regions with conserved synteny in macaque (> 75% S5 Fig), the proportion being similar to that observed for phylogenetically conserved genes. *De novo* transcripts were enriched in transposable elements; about 20% of their total transcript length was covered by transposable elements, whereas only 8% was covered in conserved genes (S6 Fig). An enrichment in transposable elements was previously observed in primate-specific protein-coding genes [12] as well as in lncRNAs in general [51].

### *De novo* genes are enriched in testis

We determined which genes were expressed in different human and chimpanzee tissues using the RNA-Seq data. The vast majority of *de novo* transcripts were expressed in testis (93.8–94.5%), as were transcripts from phylogenetically conserved genes (Fig 2d). In contrast, in brain, liver and heart, transcripts from *de novo* genes were underrepresented when compared to transcripts from conserved genes. This enrichment in testis has also been observed for mammalian lncRNAs [38,45,52]. It does not appear to be the result of increased capacity to detect weakly expressed genes in this tissue, as deduced from the overall distribution of gene expression values in testis compared to other tissues (S7 Fig). It was previously reported that young human protein-coding genes were enriched in the brain [46], but we did not detect a similar bias in our data.

As a result of the aforementioned differential expression patterns, *de novo* genes were twice as likely to show testis-restricted expression than the rest of genes (94.1%-96.4% as opposed to ~64% of all assembled transcripts, see Material and Methods). The use of gene expression data from GTEx, although limited to human annotated transcripts, produced consistent results (S8 Fig). The majority of *de novo* genes were detected in all or nearly all the 60 individuals with testis sequencing data in GTEx [53], indicating that they are expressed in a stable manner in the population (Fig 2e).

### Signatures of transcription initiation and elongation in *de novo* genes

Divergent transcription from bidirectional promoters is widespread in eukaryotic genomes [54,55] and leads to the expression of numerous transcripts in antisense orientation, most of them poorly conserved in other species and generally lacking coding potential [56]. It has been proposed that the reuse of existing promoters can be a driving force of new gene origination [57]. We searched for bidirectional promoters by scanning the genome for transcription start sites of antisense transcripts at a distance < 1 Kb. Our hits had an average distance between the two TSSs of about 100 bp, consistent with the presence of a bidirectional promoter (S9 Fig). However, *de novo* genes were not enriched in bidirectional promoters with respect to the rest of genes (20% versus 29.81%), indicating that this is not the predominant mechanism for *de novo* gene formation.

**Fig 3:**
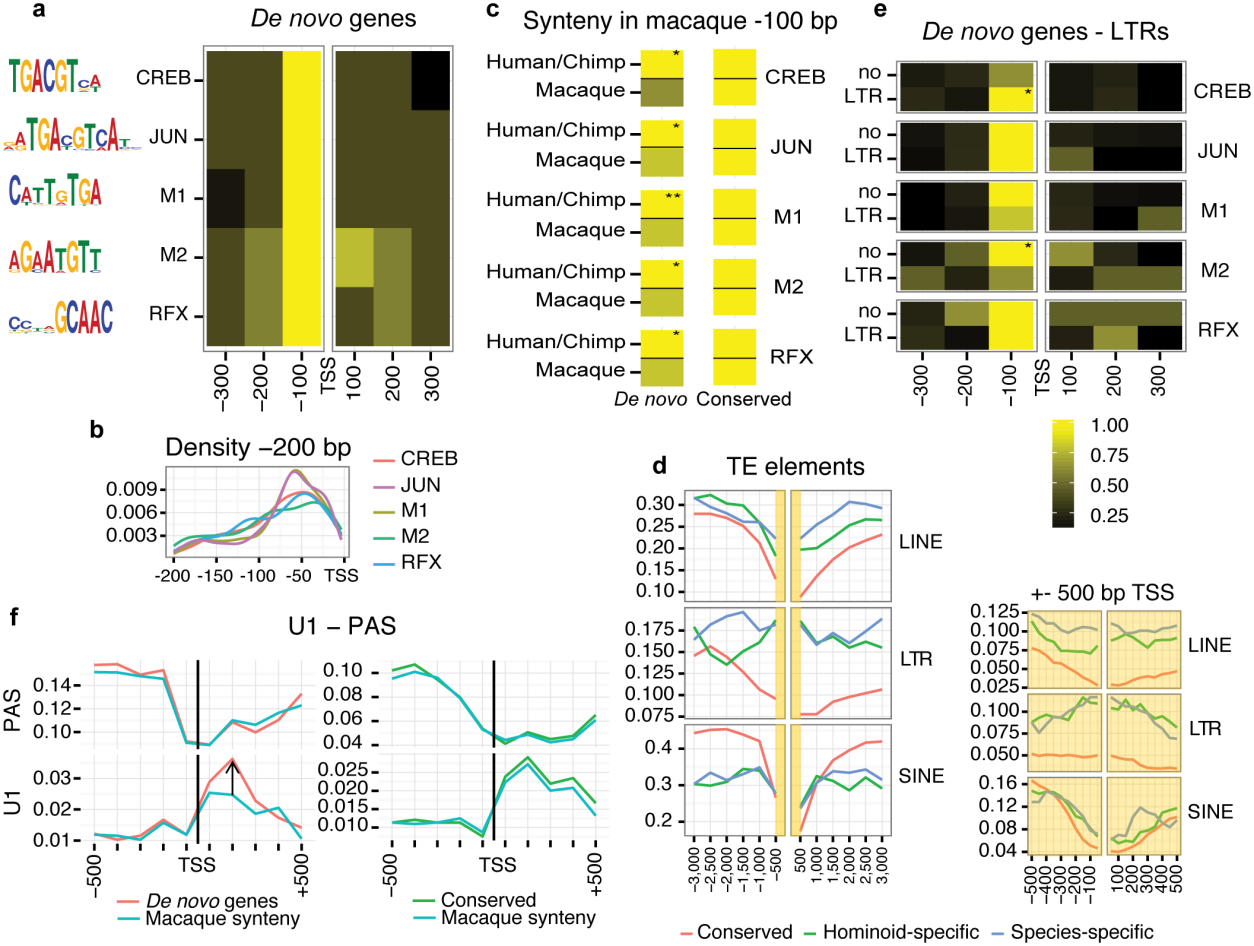
Recent signatures of transcription in *de novo* genes. **a)** Overrepresented transcription factor binding sites (TFBS) in the region -100 to 0 with respect to the transcription start site (TSS) in *de novo* genes. The region from -300 to +300 with respect to the TSS was analysed (n = 3,875). Color code relates to normalized values (highest value is yellow). **b)** Fine-grained motif density 200bp upstream of the TSS is shown. **c)** Comparison of motif density in genomic syntenic regions in macaque for *de novo* transcripts (n = 3,116) and conserved transcripts (n = 4,323, randomly taken human and chimpanzee annotated transcripts not classified as *de novo*). Significant differences between human/chimpanzee and macaque are indicated; Fisher-test; *, p-value < 0.05; **, p-value < 0.01. **d)** Density of the main human transposable elements (TE) families around the TSS of *de novo* and conserved transcripts. Regions -3 kB to +3 kB with respect to the TSS were analyzed. LTR frequency is higher in the region -100 to +100 in de novo genes when compared to conserved genes (Fisher-test p-value < 10^−18^). **e)** Comparison of motif density in promoters with and without long terminal repeat (LTR) in the region -500 to 0 with respect to the TSS. Significant differences in motif density in the -100 bp window are indicated. **f)** Signatures of transcription elongation in *de novo* and conserved genes. Density of U1 and PAS motifs in the 500bp region upstream and downstream of the TSS. Comparison of U1 and PAS motif density in genomic syntenic regions in macaque for *de novo* transcripts (n = 3,116) and conserved transcripts (n = 4,323). There is an increase of U1 motifs in *de novo* transcripts when compared to macaque (indicated by a black arrow, Fisher-test, p-value = 0.016 for the region +100 to +200).

Comparison of GC content in the region surrounding the TSSs clearly revealed that *de novo* genes are more A/T-rich than conserved annotated genes (S10 Fig). We searched for overrepresented transcription factor binding sites in the promoters of *de novo* genes using the programs PEAKS [58] and HOMER [59] (Fig 3a and 3b). With PEAKS we identified a clear enrichment of sites for CREBP, RFX, and JUN in the first 100 bp upstream of the TSS (p-value < 10^−5^, motif frequency > 20% higher than in other sequence bins). While CREBP (cAMP-responsive element binding protein) and JUN (transcription factor AP1) are general transcriptional activators, RFX (regulatory factor X) has been associated with expression in testis [60,61]. With HOMER we identified the same three motifs as well as two additional motifs (M1, M2) enriched in the first 100 bp upstream of the TSS. M1 and M2 matched the transcription factor TFIIB (RNA polymerase II complex) downstream element (BREd), which has the consensus sequence G/A-T-T/G/A-T/G-G/T-T/G-T/G [62].

We argued that, if the expression of *de novo* human and chimpanzee genes was at least partly due to the co-option of genomic sequences as active promoters, we should observe a lower frequency of the relevant TFBS in the corresponding syntenic regions in macaque. This is exactly what we found for the five motifs mentioned earlier, whereas no differences in motif frequencies existed for conserved genes (Fig 3c, S11 Fig). This was consistent with the gain of new transcription factor binding sites in the hominoid branches after the split from macaque in the *de novo* genes. We also noted that the occurrence of transposable elements (Fig 3d) tended to decrease near the TSS of all gene classes except for endogenous retrovirus-derived long terminal repeats (LTRs), which on average overlapped 13% of the proximal promoters of *de novo* genes compared to 5% in conserved genes. Further analyses indicated that LTRs tend to contribute CREB motifs (Fig 3e).

Transcription elongation is highly dependent on the presence of U1 small nuclear ribonucleoprotein recognition sites downstream of the TSS, whereas poly(A) sites (PAS) cause transcription termination [63]. The sequences bound by U1 correspond to 5’ splice sites (5’ss). As in standard multiexonic mRNAs, *de novo* genes showed enrichment of U1 sites and depletion of PAS downstream of the TSS. As U1 sites suppress the effect of PAS sites, we predicted that if transcription elongation is restricted to hominoids, we should see an underrepresentation of U1 sites in the corresponding macaque syntenic regions, but not necessarily of PAS sites. We indeed observed this pattern in *de novo* genes, whereas no differences were detected for conserved genes (Fig 3f). This is consistent with the idea that the gain of U1 sites contributes to the stabilization of *de novo* genes.

### *De novo* originated proteins

**Fig 4:**
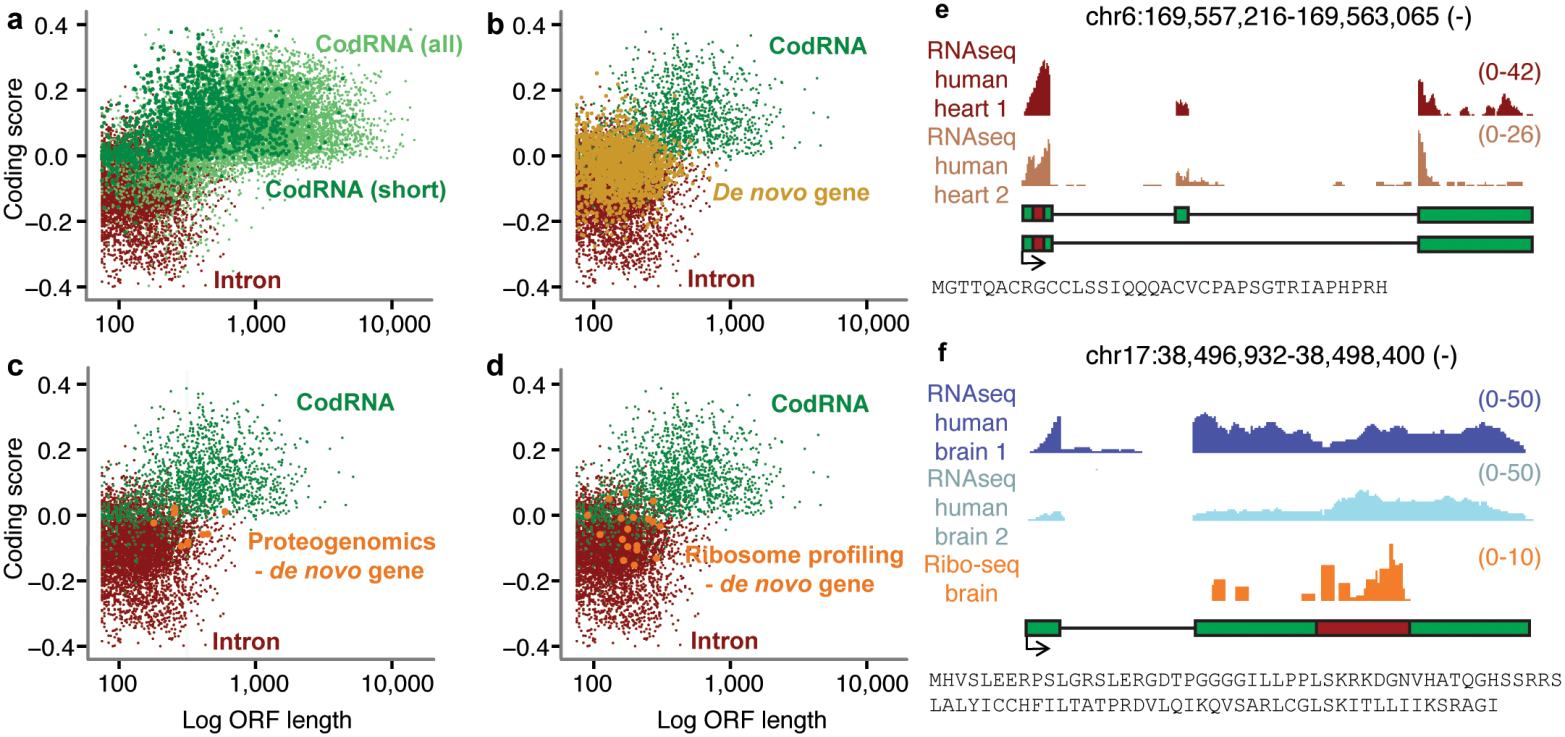
Coding potential of *de novo* genes. **a-d)** ORF length and coding score for ORFs in different sequence types. *De novo* gene, longest ORF in *de novo* transcripts (n = 1,933). CodRNA (all), annotated coding sequences from Ensembl v.75 (n = 8,462). CodRNA (short), annotated coding sequences sampled as to have the same transcript length distribution as *de novo* transcripts (n = 1,952). Intron, longest ORF in intronic sequences from annotated genes sampled as to have the same transcript length distribution as *novo* transcripts (n = 5,000); Proteogenomics—ORFs in *de novo* transcripts with peptide evidence by mass-spectrometry; Ribosome profiling—ORFs in *de novo* transcripts with ribosome association evidence in brain. **e)** Example of hominoid-specific *de novo* gene with evidence of protein expression from proteogenomics, with RNA-Seq read profiles in two human samples. **(f)** Example of hominoid-specific *de novo* gene with RNA-Seq and ribosome profiling read profiles. Predicted coding sequences are highlighted with red boxes and the putative encoded protein sequences displayed.

Most *de novo* genes were not annotated in the databases and their coding status was unclear. We analyzed two coding properties in *de nov*o genes as well as in other sequences: ORF length and ORF coding score. The latter score was based on hexanucleotide frequencies in *bona fide* sets of coding and non-coding sequences (see Methods). The median length of the longest ORF of each *de novo* gene was 52 amino acids. *De novo* predicted proteins were shorter than proteins encoded by annotated coding RNAs (codRNA) with the same transcript length distribution as the set of *de novo* genes, and comparable to ORFs from similarly sampled intronic sequences (Fig 4a and 4b). In contrast, the coding score of the longest ORF was higher in *de novo* genes than in intronic ORFs (Wilcoxon test, p-value < 10^−10^) and comparable to the score for proteins shorter than 100 amino acids in the set of annotated protein-coding genes.

Next we searched for experimental evidence of proteins produced by *de novo* genes. We employed mass-spectrometry data from a recent study [64], limiting the searches to the same tissues we used for transcript assembly to increase specificity (testis, brain, heart, and liver), and also searched in Proteomics DB [65]. We identified uniquely mapping peptides in 6 *de novo* genes; 1 human and 5 hominoid-specific genes (Table 1). All 6 were expressed in testis; one was preferentially expressed in heart. In addition, we detected signatures of translation in 5 human and 10 hominoid-specific *de novo* genes using available ribosome profiling sequencing data from human brain [66]. Overall, 21 *de novo* genes had evidence of translation.

**Table 1:** Human *de novo* genes with evidence of protein translation.

Detection Technique	Assembly gene ID	Assembly transcript ID	Age^c^	Tissue^d^	Protein length	Annotation^e^
Proteogenomics^a^	XLOC_175402	hsa_00362506	Hominoid	Heart	36	LncRNA (ENSG00000223485)
	XLOC_068697	hsa_00142705	Hominoid	Testis	37	Novel
	XLOC_085716	hsa_00181285	Hominoid	Testis	64	Novel
	XLOC_088783	hsa_00187116, hsa_00187117, hsa_00187118	Hominoid	Testis	148, 136, 61	LncRNA (ENSG00000263417)
	XLOC_105288	hsa_00223807	Hominoid	Testis	199	Novel
	XLOC_196865	hsa_00404039	Human	Testis	49	Novel
Ribosome profiling^b^	XLOC_002919	hsa_00006742, hsa_00006743, hsa_00006744	Hominoid	Brain, Heart	68, 64, 58	Novel
	XLOC_031861	hsa_00068400	Human	Brain	58	LncRNA (ENSG00000273409)
	XLOC_042102	hsa_00090118	Hominoid	Brain	90	LncRNA (ENSG00000257061)
	XLOC_050821	hsa_00107269	Human	Brain	56	Novel
	XLOC_057303	hsa_00119633	Hominoid	Testis, Brain	52	Novel
	XLOC_073846	hsa_00154236	Hominoid	Brain	54	Novel
	XLOC_082421	hsa_00173626, hsa_00173627	Hominoid	All 4 tissues	95, 95	LncRNA (ENSG00000265666)
	XLOC_085590	hsa_00181107, hsa_00181108	Hominoid	Brain,Testis	89, 83	Novel
	XLOC_104066	hsa_00221170	Hominoid	Brain	68	Novel
	XLOC_106910	hsa_00227119	Human	Brain	36	LncRNA (ENSG00000228999)
	XLOC_152506	hsa_00317537	Hominoid	Brain	53	LncRNA (ENSG00000251423)
	XLOC_160844	hsa_00333276, hsa_00333277	Hominoid	Brain	65, 65	Novel
	XLOC_168602	hsa_00348960	Hominoid	Brain	29	LncRNA (ENSG00000228408)
	XLOC_184660	hsa_00380291	Human	Brain	101	LncRNA (ENSG00000236197)
	XLOC_195038	hsa_00400469	Human	Brain	42	novel

^a^ Proteogenomics, detection is based on the identification of mass spectrometry peptides with a unique match to an ORF and corrected p-value (q-value) < 0.01 (brain, heart, liver and testis data from [64]).

^b^ Ribosome profiling, detection is based on the presence of ribosome profiling reads overlapping the ORF (brain data from [66]).

^c^Age refers to whether the gene is human-specific or hominoid-specific.

^d^The tissue with preferential expression is indicated, using the RNA-Seq data generated here for human brain, heart, liver and testis.

^e^Annotation refers to the classification of the transcripts as novel or annotated in Ensembl v.75.

Closer inspection of the genes with experimental protein evidence showed that their size (median 76 amino acids) and coding potential (median 0.0414) were in line with the values observed in the rest of *de novo* genes (Fig 4c and 4d). Specific examples of proteins encoded by *de novo* genes are shown in Fig 4e and 4f. Two thirds of the ORFs in these genes were truncated in the syntenic region in macaque and none of them were detected in the syntenic region in mouse, consistent with absence of the proteins in these species (S12 Fig). These genes showed significant signatures of purifying selection (Table 2); this was assessed by calculating the fraction of nucleotide substitutions in different gene regions (introns, exons, ORF) with respect to the corresponding macaque syntenic genomic sequences. We tested whether the sequences had a lower number of substitutions than sequences evolving in a neutral or nearly neutral manner (introns), which would indicate purifying selection. We have to consider that this is a conservative test, as selection is not expected to have acted in the macaque branch in *de novo* genes, and positive selection may increase the number of substitutions counteracting the effect of negative selection. Despite this, signatures of purifying selection could be clearly distinguished in ORFs from the *de novo* genes with evidence of translation when compared to intronic regions (Fisher-test, p-value < 0.005), as it occurs in coding sequences encoding functional proteins (Table 2). In contrast, in *de novo* genes in general there was not a significant decrease in the number of substitutions in the longest ORF when compared to neutrally evolving sequences, suggesting that the majority of these transcripts do not encode functional proteins.

**Table 2:** Divergence with macaque syntenic regions. Estimated number of substitutions per Kb (PAML). Dataset 3 corresponds to the genes in Table 1. ORF in datasets 1 and 2 is the longest ORF in the transcript. Introns refers to sampled intronic regions of size 500 bp from the same set of transcripts. We tested for differences between complete exons and introns, and ORF and introns with the Fisher test.

	Transcript			Introns
Dataset	Complete exons	ORF	Rest exonic sequence	
1. Species-specific *de novo* transcripts	70.20 ***	71.6	69.84	72.51
2. Hominoid-specific *de novo* transcripts	69.80 ***	72.9	69.13	71.82
3. *De novo* transcripts with protein evidence	61.75 ***	49.75 **	64.75	71.4
4. Conserved annotated transcripts	36.30 ***	26.90 ***	37.99	72.15

*p-value < 0.05,

**p-value<0.005,

***p-value < 10^−5^.

## Discussion

We performed a large-scale transcriptomics-based investigation on the emergence of new genes in hominoids. Our strategy was annotation-independent, which allowed us to recover many novel (non-annotated) genes and compare species for which the level of annotation varies greatly. The approach was entirely different from that employed in previous studies in which the initial datasets were composed of annotated protein coding genes in humans that lacked homologous proteins in other species [12,19–21]. We instead focused on new transcriptional events and subsequently analyzed the properties of the transcripts including coding potential and purifying selection signatures. We assembled the transcriptomes from different species to account for differences in the level of annotation, being able to recover a large number of genes likely to have originated very recently.

We employed a polyadenylated RNA sequencing strategy that was based on a combination of high sequencing depth and strand-specific sequencing, with an average of 115 Million mapped reads per sample. After performing exhaustive sequence similarity searches, we identified 2,714 genes which were specific of human, chimpanzee, or their hominoid ancestor. This is more than one order of magnitude greater than the number of human or primate-specific genes reported in previous studies [12,19–21]. The *de novo* origin of these genes is supported by the lack of genes expressed in the corresponding syntenic genomic regions of closely related species. We employed a carefully chosen per-base read coverage threshold, which allowed for the full recovery of complete sequences while permitting the detection of transcripts which were expressed at low levels. Our analysis was based on multiexonic genes but we have to consider that many recently evolved genes may not have yet acquired the capacity to be spliced, as shown by several examples in *Drosophila* [41]. Therefore, there are probably many more *de novo* genes than those studied here. The *de novo* genes constituted about 4% of all expressed multiexonic genes in human and chimpanzee. This fraction is consistent with similar transcriptomics-based studies in insects [40,24]. As these genes are short and expressed at low levels, their associated transcriptional cost is relatively small. *De novo* genes showed characteristic promoter and splicing signals and were expressed in a consistent manner across different individuals. However, they had very weak purifying selection signatures in general. This is interesting because it means that even if these genes are expressed in a stable manner, many of them are likely to lack functionality and thus can be considered protogenes [33].

The proportion of *de novo* genes with conserved genomic synteny in macaque was comparable to that of conserved genes. Given the low number of nucleotide differences in neutrally evolving regions between these two species (~ 6%), we could reliably use syntenic alignments to examine transcription-related sequence features. Relative to the corresponding genomic regions in macaque, we found an enrichment of transcription factor binding sites and U1snRNP motifs in *de novo* genes in human and chimpanzee; this is consistent with the idea that the gain of regulatory motifs underlies *de novo* gene origination. This scenario had been proposed for the formation of a new gene in mouse [7,10] but until now it had not been considered at a genome-wide scale. Interestingly, in addition to general activators and polymerase II binding sites we found an enrichment in RFX motifs in *de novo* gene promoters. Although there are several members of the RFX transcription factor family that bind to similar sequences, many of the sites in our sequences may be recognized by RFX2, which is highly expressed in testis and has been involved in spermiogenesis [61].

Several studies have found an excess of genes of very recent origin when compared to older gene classes [40,24]. This suggests that many young genes are subsequently lost, which is consistent with the relatively constant number of genes observed in a taxon. Our finding that signatures of purifying selection are generally very weak for *de novo* genes is indeed consistent with a scenario in which many of these genes are dispensable. However, a subset of genes with evidence of translation do display significant signatures of purifying selection, indicating that they correspond to functional genes. Studies in *Drosophila* indicate that directional selection determines the fate of some *de novo* genes from the very early stages [41]. While we focused primarily on possible coding functions, some of the genes may have also acquired non-coding functions. This is especially relevant in the case of antisense transcripts which can potentially influence the expression of the transcript in the opposite orientation [67]. It is important to consider that the annotations alone may not suffice to differentiate between coding and non-coding transcripts, as many annotated lncRNAs may translate short peptides according to ribosome profiling data [34,36,37]. LncRNAs tend to have small open reading frames and display limited phylogenetic conservation [37,68] and it has been previously proposed they may act as precursors of new protein-coding genes [13,21,37]. An interesting observation was that the coding score of *de novo* genes was clearly non-random. One possible explanation is that natural selection rapidly eliminates transcripts that produce toxic peptides [35], as one could expect such peptides to often have unusual amino acid compositions.

Here we detected 20 putative new human proteins using ribosome profiling from brain tissue [66]. Considering that the expression of most *de novo* genes was restricted to testis for which no ribosome profiling data has yet been published, we expect this number to increase substantially in the future. Mass-spectrometry has important limitations for the detection of short peptides [69], but we could nevertheless detect 8 putative proteins, mostly from testis. Our results indicate that the expression of new loci in the genome takes place at a very high rate and is probably mediated by random mutations that generate new active promoters. These newly expressed transcripts would form the substrate for the evolution of new genes with novel functions.

## Materials and Methods

### Ethics statement

Chimpanzee and macaque samples were obtained from the Primate Bio-Bank of the Biomedical Primate Research Center (BPRC). BPRC offers state-of-the-art animal facilities (AAALAC accredited) and is fully compliant with regulations on the use of non-human primates for medical research. BPRC's Primate Tissue Bank is one of the biggest non-human primate banks in Europe and it is involved in the framework of the EuprimNet Bio-Bank (www.euprim-net.eu). The EUPRIM-Net Bio-Bank is conducted and supervised by the scientific government board along all lines of EU regulations and in harmonization with Directive 2010/63/EU on the Protection of Animals Used for Scientific Purposes. The animals used for tissue collection in all cases are diagnosed with cause of death other than their participation in this study and without any relation to the tissues used.

### Library preparation and strand-specific polyA+ RNA-Seq protocol

Human and mouse total RNA was purchased from Amsbio. Chimpanzee and macaque total RNA was extracted using a miRNeasy Mini kit from tissue samples obtained at the Biomedical Primate Research Centre (BPRC, Netherlands). Mouse samples were from a pool of 3 males and 3 females (Balb/C strain).

Libraries were prepared using the TruSeq Stranded mRNA Sample Prep Kit v2 according to the manufacturer’s protocol. PolyA+ RNA was purified from 250–500 mg of total RNA using streptavidin-coated magnetic beads (AMPure XP) and subsequently fragmented to ~300 bp. cDNA was synthesized using reverse transcriptase (SuperScript II, Invitrogen) and random primers. We did not add reverse transcriptase to one of the human testis replicate samples to use it as a control for DNA contamination (RT-). The strand-specific RNA-Seq library preparation was based on the incorporation of dUTP in place of dTTP in the second strand of the cDNA. Double-stranded DNA was further used for library preparation. Such dsDNA was subjected to A-tailing and ligation of the barcoded Truseq adapters. Library amplification was performed by PCR on the size selected fragments using the primer cocktail supplied in the kit. Sequencing was done with an Illumina HiSeq 2000 sequencer in a paired-end configuration (2x100 nt) according to the manufacturer’s instructions. Library preparation and sequencing were done at the Genomics Unit of the Center for Regulatory Genomics (CRG, Barcelona, Spain).

### RNA-Seq datasets

The polyA+ RNA-Seq included 96 sequencing datasets for 9 different species: 43 strand-specific paired end data (~3 billion reads) and 53 single read data (~3.2 billion reads). The strand-specific data was employed for the assembly of reference transcripts for human, chimpanzee, macaque, and mouse (Fig 1 for a summary of results). For comparative purposes, we used the same tissues and number of biological samples for human and chimpanzee (liver, heart, brain, and testis; two biological replicates per tissue). For macaque and mouse, we added available strand-specific RNA-Seq data from other tissues: adipose, skeletal muscle for macaque [20], and ovary and placenta for mouse [38,45]. The single read data corresponded to 5 primate species (human, chimpanzee, gorilla, orangutan, and macaque) and 4 additional vertebrates (mouse, chicken, platypus, and opossum) in 6 different tissues (brain, cerebellum, heart, kidney, liver, and testis) [50]. While these experiments were based on single reads and had lower coverage than the strand-specific RNA-Seq data, they were used to increase the number of species with expression data for sequence similarity searches. More information about the samples can be found in S1 Dataset. Sequencing data generated for this study is deposited in the Gene Expression Omnibus under accession number GSE69241.

### Read mapping and transcriptome assembly

RNA-Seq sequencing reads underwent quality filtering using Condetri (v.2.2) [70] with the following settings (-hq = 30 –lq = 10). Adapters were trimmed from filtered reads if at least 5 nucleotides of the adaptor sequence matched the end of each read. In all experiments, reads below 50 nucleotides or with only one member of the pair were not considered. We retrieved genome sequences and gene annotations from Ensembl v. 75 [71]. We aligned the reads to the correspondent reference species genome with Tophat (v. 2.0.8) [72] with parameters –N 3, -a 5 and –m 1, and including the correspondent parameters for paired-end and strand-specific reads whenever necessary. Multiple mapping to several locations in the genome was allowed unless otherwise stated.

We performed gene and transcript assembly with Cufflinks (v 2.2.0) [44] for each individual sample. Per-base read coverage and FPKM (fragments per kilobase of transcript per million mapped fragments) values were calculated for each transcript and gene as described by [44]. We only considered assembled transcripts that met the following criteria: a) the transcript was covered by at least 4 reads, b) Abundance was higher than 1% of the most abundant isoform of the gene and, c) <20% of reads were mapped to multiple locations in the genome.

Subsequently, we used Cuffmerge [44] to build a single set of assembled transcripts for each species, always keeping the strand-specific and the single read based RNA-Seq experiments separate. We compared our set of assembled transcripts with gene annotation files from Ensembl (gtf format, v.75) with Cuffcompare [44] to identify transcripts corresponding to annotated genes. This included the categories ' = ' (complete match), 'c' (contained), 'j' (novel isoform), “e”, and “o” (other exonic overlaps in the same strand). Genes for which none of the assembled transcripts matched an annotated gene were labeled ‘novel’. In human, 82% of the total annotated protein-coding and 44.5% of the non-coding genes (lincRNA, antisense and processed transcripts) were recovered.

Additionally, we ran Trinity [47], which reconstructs transcripts in the absence of a reference genome, with all unmapped reads in each species (read length > = 75 nucleotides). Before running Trinity, unmapped reads were normalized by median using Khmer (parameters –C 20, -k 20, -N 4). This allowed the recovery of any transcripts falling into non-assembled parts of the genome. We selected transcripts with a minimum size of 300 nucleotides.

We obtained a set of reference transcripts from the strand-specific RNA-Seq data using a per-nucleotide read coverage > = 5. This choice was based on the relationship between read coverage and the percentage of fully reconstructed annotated coding regions (CDS, longest one per gene) for the subset of genes mapping to annotated protein coding genes (Ensembl v.75) using only the categories ' = ' and 'c' in Cuffcompare (18,694 protein-coding genes). For values higher than 5 there was no substantial increase in the percentage of fully reconstructed CDS (coverage > = 5: 87.8%; coverage > = 10: 88.5%; coverage > = 20: 89.4%). The selection was based on coding regions and not complete transcripts because of the prevalence of alternative transcription start sites in many annotated transcripts, causing uncertainty in the latter parameter [73]. Very similar results were obtained for CDS shorter than 500 nucleotides or genes with only one annotated CDS, indicating that protein length or gene complexity has little effect on the suitability of this threshold.

Transcript assembly with the RT- control (see above) resulted in 22,803 different sequences that presumably corresponded to genomic DNA contamination, resulting from regions resistant to DNAse treatment. Except for the reverse transcriptase, all other reagents were added in the same concentration as in the other samples. Therefore, the number of contaminant fragments must be considered an upper boundary, as in a normal RNA-Seq experiment these fragments are probably sequenced much less efficiency as they have to compete with the genuine RT products. The sequences obtained in the RT- control did not contain any introns and the majority of them were shorter than 300 nucleotides (98.58%).

### Genomic comparisons

Reference transcripts were classified into three categories depending on their location with respect to transcripts from other genes: a) Intergenic: Transcripts that did not overlap any other assembled locus. b) Overlapping intronic: Transcripts located within introns of other assembled genes on the opposite strand. c) Overlapping antisense: Transcripts partially or completely overlapping exons from other assembled genes on the opposite strand.

We downloaded long interspersed element (LINE), short interspersed element (SINE), and long terminal repeat (LTR) annotations in the human and chimpanzee genomes from RepeatMasker (same genome versions than in Ensembl v.75) [74]. We used BEDTools [75] to identify any overlap between transcripts and/or genomic elements.

We downloaded human-chimpanzee, human-macaque, human-mouse, chimpanzee-macaque and chimpanzee-mouse pairwise syntenic genomic alignments, obtained by blastz [76], from UCSC. We developed an in-house Python script to recover syntenic regions corresponding to a given human or chimpanzee transcript, or to regions upstream and downstream of a human or chimpanzee transcription start site (TSS), using these alignments.

We scanned the human and chimpanzee genomes to identify transcripts with bidirectional promoters. We recovered any antisense pairs in which the distance between the two TSSs was < 1 kb). We estimated that 29.81% of the conserved genes and 20% for *de novo* genes were expressed from bidirectional promoters. This was significantly higher than the number expected by chance (5,31%, Binomial Test, p-value << 10^−5^). The location of different types of genes in the human chromosomes was visualized with Circos [77].

### Identification of *de novo* genes

We developed a pipeline to identify *de novo* genes in human and chimpanzee based on the lack of homologues in other species. We first selected multiexonic transcripts from the reference transcriptome assemblies. Then, we performed exhaustive sequence similarity searches against sequences from other species with the BLAST suite of programs. Subsequently, we searched for overlapping transcripts in genomic syntenic regions.

Sequence similarity searches, using reference human or chimpanzee transcripts as query, were performed against the complete transcriptome assemblies from the nine different vertebrate species, gene annotations from Ensembl v.75 for the same species, and the EST and non-redundant protein “nr” [78] NCBI databases. We employed both BLASTN and TBLASTX programs [49], with an E-value threshold of 10^−4^. All BLAST searches were performed with the filter of low-complexity regions activated; we discarded all transcripts for which self-hits were not reported. Species-specific genes were those for which no transcripts (or transcripts of any paralogs) had sequence similarity hits to transcripts in any other species. To identify synteny-based homologues we took advantage of the existing pairwise syntenic genomic alignments from UCSC. We used data from human, chimpanzee, macaque, and mouse. If two transcripts overlapped (> = 1bp) in a syntenic region we considered it as evidence of homology. We reclassified the *de novo* genes accordingly.

We identified 634 human-specific genes (1,029 transcripts) and 780 chimpanzee-specific genes (1,307 transcripts). In the case of hominoid-specific genes we allowed for hits to gorilla and orangutan in addition to human and chimpanzee; this yielded 1,300 hominoid-specific genes (3,062 transcripts). About one third of them (221 genes and 1,016 transcripts) were reference transcripts in both species (multiexonic, coverage > = 5) and the rest were identified via the complete transcriptome assemblies, EST, and/or nr databases. Due to the fact that not all of these genes were detected as reference transcripts in both species the number of hominoid-specific genes is different for human and chimpanzee (604 and 916, respectively). Annotation files of *de novo* genes in GTF format are available at Figshare, http://dx.doi.org/10.6084/m9.figshare.1604892 (human) and http://dx.doi.org/10.6084/m9.figshare.1604893 (chimpanzee).

### Tissue gene expression

We analyzed the patterns of tissue expression in assembled transcripts, considering a transcript as expressed in one tissue if FPKM > 0. We measured the number of tissue-restricted transcripts using a previously proposed metric [79]:

$$\tau =\frac{\sum_{i=1}^{i=n}\left(1-x_{i}\right)}{n-1}$$

Where n is the number of tissues and x_i_ is the FPKM expression value of the transcript in the sample normalized by the maximum expression value over all tissues. We classified cases with a τ > 0.85 as preferentially expressed in one tissue or as tissue-restricted.

For *de novo* genes annotated in Ensembl v.75 we obtained expression data from the GTEx project, which comprises a large number of human tissue samples. We used this data to calculate the number of genes showing tissue-restricted expression as well as the number of testis samples with detectable expression of a given gene.

### Motif analysis

We searched for significantly overrepresented motifs in *de novo* and conserved genes using computational approaches. We employed sequences spanning from 300 bp upstream to 300 bp downstream of the transcription start site (TSS). Redundant TSS positions were only considered once. With PEAKS [58] we identified three TRANSFAC motifs [80] enriched in *de novo* genes, corresponding to CREB, JUN, RFX. HOMER [59], a tool for motif discovery, also detected these motifs plus two additional motifs (M1, M2). The five motifs were enriched in the first 100 bp upstream of the TSS (p-value < 10^−5^, minimum 30 motif occurrences and enrichment > 20% when compared to other regions). M1 and M2 matched the transcription factor TFIIB (RNA polymerase II complex) downstream element (BREd), which has the consensus sequence G/A-T-T/G/A-T/G-G/T-T/G-T/G [62].

For graphical representation of the results, we computed the relative motif density in 100 bp windows upstream and downstream of the TSS in human and chimpanzee, and the corresponding genomic syntenic regions in macaque and mouse. We used MEME [81] to scan the sequences for the occurrence of motifs (matches to weight matrices with a p-value < 10^−5^). The average number of motif occurrences (motif density) was normalized to values between 0 and 1, where 1 corresponded to the highest density of a given motif in a sequence window.

It has been previously proposed that new genes tend to gain new U1 sites and lose PAS sites as they become more mature [63]. We used MEME with the same parameters as described above to search for U1 (U1 snRNP 5’ splice site consensus motif) and PAS (poly-adenylation signals) sites 500 bp upstream and downstream of the TSS (see supplementary material for weight matrices). PAS motifs found < 500bp downstream of a U1 site were not considered since the PAS effect is abolished by snRNPs bound to these U1 motifs at such distances.

### Coding score

We defined an open reading frame (ORF) in a transcript as any sequence starting with an ATG codon and finishing at a stop codon (TAA, TAG or TGA). In addition we require it to be at least 75 nucleotides long (24 amino acids), which is the size of the smallest complete human polypeptide found in genetic screen studies [82].

In each ORF we computed a coding score based on hexamer frequencies in *bona fide* coding and non-coding sequences [37]. Specifically, we first computed one coding score (CS) per nucleotide hexamer:

$$CS_{hexamer(i)}=\text{log}\frac{freq_{coding}(hexamer(i))}{freq_{non-coding}(hexamer(i))}$$

The coding hexamer frequencies were obtained from all human transcripts encoding experimentally validated proteins. The non-coding hexamer frequencies were calculated using the longest ORF in intronic regions which were selected randomly from expressed protein-coding genes. The hexamer frequencies were computed separately for ORFs with different lengths to account for any possible length-related biases (24–39, 40–59, >60 amino acids). Next, we used the following statistic to measure the coding score of an ORF:

$$CS_{ORF}=\frac{\sum_{i=1}^{i=n}CS_{hexamer(i)}}{n}$$

Where i is each hexamer sequence in the ORF, and n is the number of hexamers considered.

The hexamers were calculated in steps of 3 nucleotides in frame (dicodons). We did not consider the initial hexamers containing a Methionine or the last hexamers containing a STOP codon. Given that all ORFs were at least 75 nucleotides long, the minimum value for n was 22.

In coding RNAs (CodRNA all) the annotated ORF was selected for further analysis. To account for any possible bias due to transcript length, we randomly selected a subset of protein-coding transcripts (CodRNA short) with the same transcript length distribution as the *de novo* transcripts. In sequences with no annotated coding sequence (introns and transcripts from *de novo* genes), we chose the longest ORF considering all three possible frames. The only exception was when the longest ORF in another frame had a higher coding score than expected for non-coding sequences (0.0448 if ORF < 40 aa; 0.0314 if 60 aa > length ORF > = 40 aa; 0.0346 if length ORF > = 60 aa; p-value < 0.05) or if it was longer than expected for non-coding sequences (> = 134 aa, p-value < 0.05). In this very small number of cases (3.4%) we selected this other ORF.

### Ribosome profiling data

We downloaded data from ribosome profiling experiments in human brain tissue [66]. Ribosome profiling reads were filtered as described previously [37]. We then used Bowtie2 [83] to map the reads to the human assembled transcripts with no mismatches. We considered each strand independently since the RNA-Seq data was strand-specific. RNA-Seq reads from the same experiment were also mapped to *de novo* transcripts to determine how many of them were expressed (FPKM > 0). Because of the low detectability of ribosome association at low FPKM expression values [37], two ribosome profiling reads mapping to a predicted ORF were deemed sufficient for the signal to be reported.

### Mass spectrometry data

We used available mass-spectrometry data from human frontal cortex, liver, heart, and testis [64,65] to identify any putative peptides produced by *de novo* genes. Mass-spectrometry data was analyzed using the Proteome Discoverer software v.1.4.1.14 (Thermo Fisher Scientific, United States) using MASCOT v2.5 [84] as a search engine. The database we used contained the human entries in SwissProt [85], the most common contaminants, and putative peptides derived from the translation of transcripts from *de novo* genes. Carbamidomethylation for cysteines was set as fixed modification whereas acetylation in protein N-terminal and oxidation of methionine were set as variable modifications. Peptide tolerance was 7 ppm in MS and 20mmu in MS/MS mode, maximum number of missed cleavages was set at 3. The Percolator [86] algorithm implemented in the Proteome Discoverer software was used to estimate the qvalue and only peptides with qvalue < 0.01 and rank = 1 were considered as positive identifications. Lastly, we considered unique peptides matching young transcripts by using BLAST with short query parameters to search the candidate peptides against all predicted ORFs in assembled transcripts. Additionally, we searched for any matching peptides in Proteomics DB [65]. We found 6 *de novo* genes with proteomics evidence; two of them were annotated in Ensembl as lncRNAs and expressed in ≥55 testis samples from GTEx. Details of the results can be found in the supplementary material.

### Calculation of substitution rates

We estimated the number of substitutions per Kb in human-macaque genomic alignments with the maximum likelihood method ‘baseml’ from the PAML package [87] with model 4 (HKY85). We only analyzed transcripts with complete synteny in both species. We compared the number of substitutions with respect to sequence length in different sequence sets using the Fisher exact test.

### Statistical data analyses and plots

The analysis of the data, including generation of plots and statistical test, was done using R [88].

## Supporting Information

S1 Fig Comparison of human genes assembled in this study and in other published datasets. 'Ruiz-Orera' is this study. 'Necsulea' represents genes that match lncRNAs annotated in “Necsulea A, Soumillon M, Warnefors M, Liechti A, Daish T, et al. (2014) The evolution of lncRNA repertoires and expression patterns in tetrapods. Nature 505: 635–640” [38]. 'Iyer' refers to genes that match lncRNAs annotated in “Iyer MK, Niknafs YS, Malik R, Singhal U, Sahu A, et al. (2015) The landscape of long noncoding RNAs in the human transcriptome. Nat Genet 47: 199–208” [48].
(PNG)

S2 Fig Summary of the filters applied to obtain the final list of *de novo* genes specific of human or chimpanzee. Transcript homology: genes discarded because of homology to transcriptomes (assemblies or annotations) from other species using sequence similarity searches. Synteny: genes discarded because they overlapped other transcripts in genomic syntenic regions. EST/nr: genes discarded because they matched sequences from the EST or nr databases.
(PNG)

S3 Fig Circos plot showing the distribution of different types of sequences in the human chromosomes. *De novo* genes include both human- and hominoid-specific genes. Pseudogenized retrocopies correspond to genes annotated as “processed pseudogenes” in Ensembl.
(PNG)

S4 Fig Properties of *de novo* transcripts when compared to all annotated and novel transcripts. **a)** Cumulative density of length in species-specific, hominoid-specific, annotated and novel assembled transcripts. **b)** Log2 cumulative density of expression values in species-specific, hominoid-specific, annotated and novel assembled transcripts. Expression is measured in fragments per kilobase per million mapped reads (FPKM) values, selecting the maximum value across all samples. Collectively, *de novo* genes had a median size of 595 nucleotides and median expression of 0.31 FPKM. Species-specific transcripts are significantly shorter (Wilcoxon test, p-value <10^−16^) than hominoid-specific transcripts, but no differences in expression levels are observed.
(PNG)

S5 Fig Conservation of syntenic genomic regions corresponding to *de novo* or conserved genes. The existence of full or partial synteny was assessed using pairwise genomic alignments from UCSC. Hominoid (inner circle) refers to human when chimpanzee is the reference species and to chimpanzee when human is the reference species. The proportion of *de novo* and conserved transcripts with full or partial synteny decreases with phylogenetic distance. The proportion of transcripts from *de novo* genes with complete genomic synteny in macaque was comparable to that of transcripts from conserved genes.
(PNG)

S6 Fig *De novo* genes are enriched in transposable elements. Transcrips covered by transposable elements (TEs) considering all annotated transcripts, hominoid-specific genes or species-specific genes (human- or chimpanzee-specific genes). CDS is the annotated coding sequence in annotated protein-coding transcripts and the longest ORF in *de novo* transcripts. Classes of TEs: LINEs; long interspersed elements; LTRs, long terminal repeats; SINEs, short interspersed elements. **a)** Average fraction of transcript length covered by TEs. **b)** Number of transcripts covered by TEs (> = 1bp overlap).
(PNG)

S7 Fig Distribution of expression values in assembled genes across tissues. Log10 cumulative density of expression values in assembled genes. Expression is measured in fragments per kilobase per million mapped reads (FPKM) values, selecting the maximum value across all samples. Testis does not show a lack of highly expressed transcripts (actually the opposite is observed for human) that could explain why we detect so many transcripts being expressed in this tissue.
(PNG)

S8 Fig Human annotated transcripts from *de novo* genes are enriched in testis according to GTEx data. Data is for annotated transcripts in the GTEx catalog which are preferentially expressed in one tissue (tissue-restricted), as measured by a tissue preferential expression index higher than 0.85 (see Methods online for more details on this index).
(PNG)

S9 Fig Distance between the transcription start site (TSS) of transcripts from *de novo* genes and the nearest TSS from another transcript, for genes with divergent transcription. These were defined as antisense genes with the TSSs separated by less than 1 kb, potentially sharing a bidirectional promoter. Negative values imply overlap between the transcripts. There is a strong peak at around 100 nucleotides.
(PNG)

S10 Fig *De novo* genes have a low GC content when compared to conserved annotated genes. Nucleotide frequencies 300 bp upstream and 300 downstream of the transcription start site (TSS) were calculated for different sets of transcripts. Conserved: 4,323 randomly taken human and chimpanzee annotated transcripts not classified as *de novo*.
(PNG)

S11 Fig Regulatory motif frequencies around the TSS. **a)** Number of matches of overrepresented motifs in 100 bp windows in *de novo* genes and in the corresponding macaque syntenic regions (corresponds to Fig 3a in main manuscript file). **b)** Same data for conserved annotated genes. **c)** Relative motif frequencies in *de novo* genes including motifs overrepresented in conserved annotated genes in general but not in *de novo* genes (NRF, MAZ, EGR-1, E2F). **d)** Data for the same motifs for conserved annotated genes.
(PNG)

S12 Fig Conservation of ORFs in syntenic genomic regions corresponding to *de novo* genes with experimental evidence of translation. The existence of full or partial synteny was assessed using pairwise genomic alignments from UCSC. Hominoid (inner circle) refers to human when chimpanzee is the reference species and to chimpanzee when human is the reference species. Only ORFs in *de novo* genes with evidences of preoteogenomics or ribosome profiling are displayed. Non-truncated ORFs are the ones in which the frame, the start codon and the stop codon are conserved in the other syntenic genomic region; otherwise the ORF is truncated.
(PNG)

S1 Table De novo genes annotated as protein-coding in Ensembl v. 75. Identification of annotated genes in the set of de novo genes was based on the comparison of the genomic coordinates of the assembled transcripts and the genomic coordinates of annotated genes using Cuffcompare. All these genes were hominoid-specific (expressed both in human and chimpanzee). (*) refers to the same orthologous gene in human and chimpanzee. Note that all human coding genes had been annotated as different classes of long non-coding RNAs (lncRNAs) in Ensembl v. 77.
(DOC)

S1 Dataset Samples and sequence data. It contains five different datasheets (T1-T5). T1. Detailed information on the RNA-Seq samples from this study. T2. Stranded assemblies, information on the transcript assemblies obtained using strand-specific RNA-Seq data. T3. Single assemblies, information on the transcript assemblies obtained using single read RNA-Seq data. T4. Weight matrices, relative nucleotide frequencies of the motif weight matrices used in this study. T5. Mass spectrometry, information on the peptides identified by proteomics.
(XLS)
