# Supplementary figures and images for: Origins of De Novo Genes in Human and Chimpanzee
Source: PLoS Genet. 2015 Dec 31;11(12):e1005721. doi: 10.1371/journal.pgen.1005721 (PMC4697840; doi:10.1371/journal.pgen.1005721)

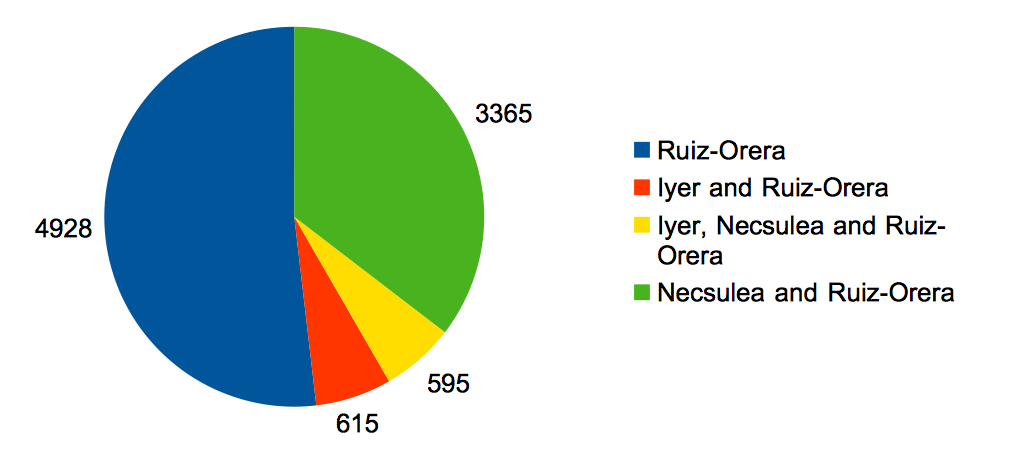

Supplement: S1 Fig — 'Ruiz-Orera' is this study. 'Necsulea' represents genes that match lncRNAs annotated in “Necsulea A, Soumillon M, Warnefors M, Liechti A, Daish T, et al. (2014) The evolution of lncRNA repertoires and expression patterns in tetrapods. Nature 505: 635–640” [38]. 'Iyer' refers to genes that match lncRNAs annotated in “Iyer MK, Niknafs YS, Malik R, Singhal U, Sahu A, et al. (2015) The landscape of long noncoding RNAs in the human transcriptome. Nat Genet 47: 199–208” [48]. (PNG) [file pgen.1005721.s001.png]

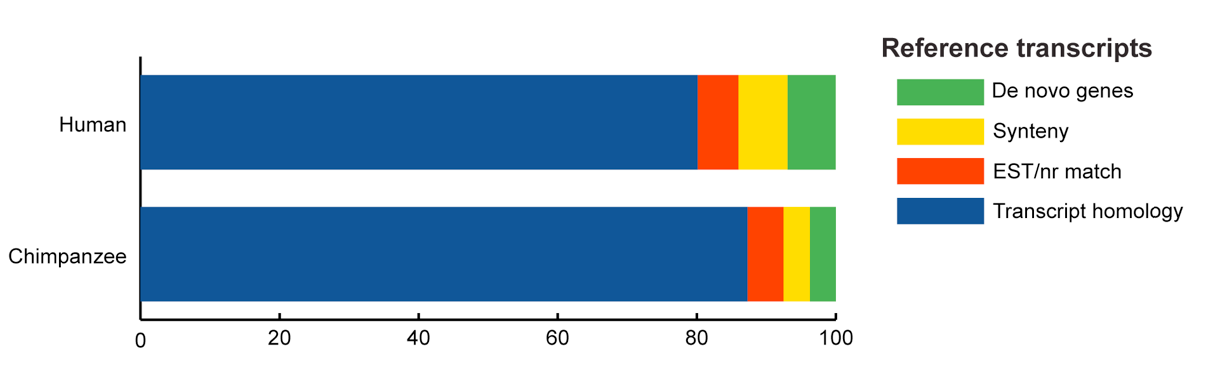

Supplement: S2 Fig — Transcript homology: genes discarded because of homology to transcriptomes (assemblies or annotations) from other species using sequence similarity searches. Synteny: genes discarded because they overlapped other transcripts in genomic syntenic regions. EST/nr: genes discarded because they matched sequences from the EST or nr databases. (PNG) [file pgen.1005721.s002.png]

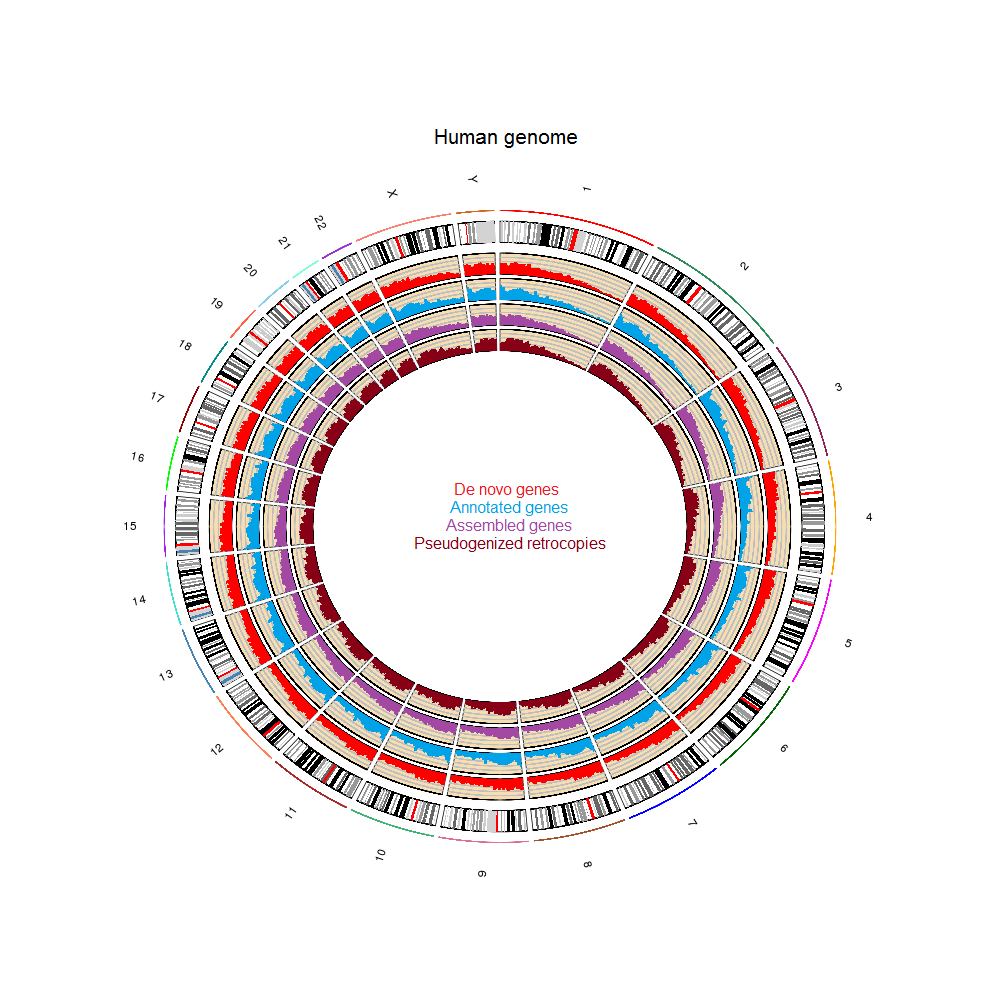

Supplement: S3 Fig — De novo genes include both human- and hominoid-specific genes. Pseudogenized retrocopies correspond to genes annotated as “processed pseudogenes” in Ensembl. (PNG) [file pgen.1005721.s003.png]

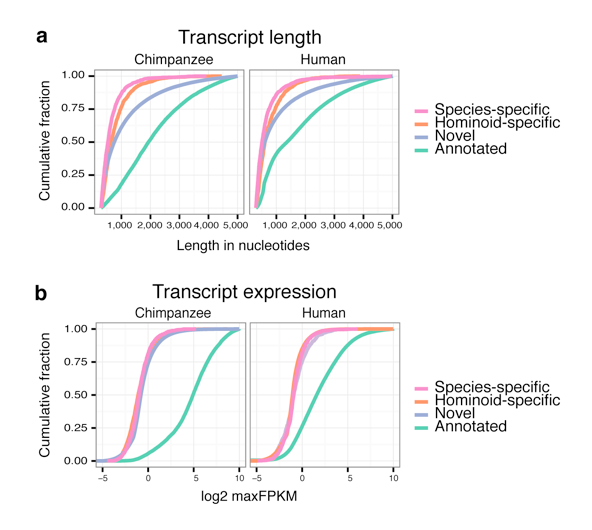

Supplement: S4 Fig — a) Cumulative density of length in species-specific, hominoid-specific, annotated and novel assembled transcripts. b) Log2 cumulative density of expression values in species-specific, hominoid-specific, annotated and novel assembled transcripts. Expression is measured in fragments per kilobase per million mapped reads (FPKM) values, selecting the maximum value across all samples. Collectively, de novo genes had a median size of 595 nucleotides and median expression of 0.31 FPKM. Species-specific transcripts are significantly shorter (Wilcoxon test, p-value <10−16) than hominoid-specific transcripts, but no differences in expression levels are observed. (PNG) [file pgen.1005721.s004.png]

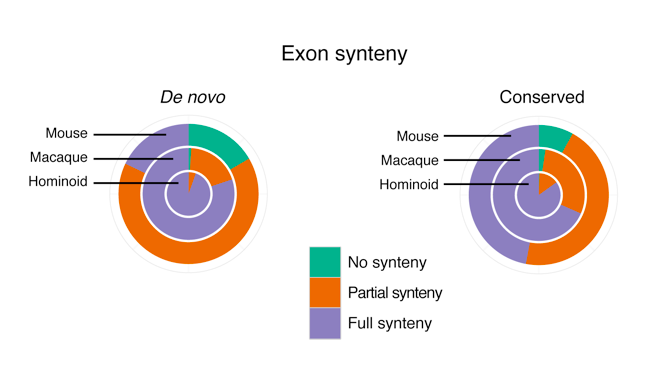

Supplement: S5 Fig — The existence of full or partial synteny was assessed using pairwise genomic alignments from UCSC. Hominoid (inner circle) refers to human when chimpanzee is the reference species and to chimpanzee when human is the reference species. The proportion of de novo and conserved transcripts with full or partial synteny decreases with phylogenetic distance. The proportion of transcripts from de novo genes with complete genomic synteny in macaque was comparable to that of transcripts from conserved genes. (PNG) [file pgen.1005721.s005.png]

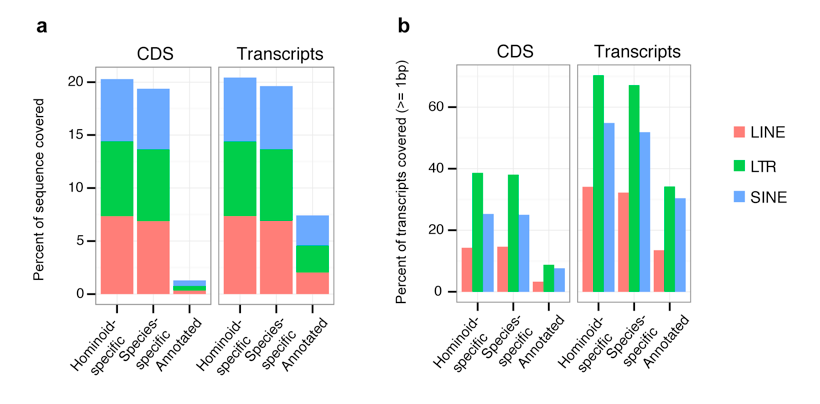

Supplement: S6 Fig — Transcrips covered by transposable elements (TEs) considering all annotated transcripts, hominoid-specific genes or species-specific genes (human- or chimpanzee-specific genes). CDS is the annotated coding sequence in annotated protein-coding transcripts and the longest ORF in de novo transcripts. Classes of TEs: LINEs; long interspersed elements; LTRs, long terminal repeats; SINEs, short interspersed elements. a) Average fraction of transcript length covered by TEs. b) Number of transcripts covered by TEs (> = 1bp overlap). (PNG) [file pgen.1005721.s006.png]

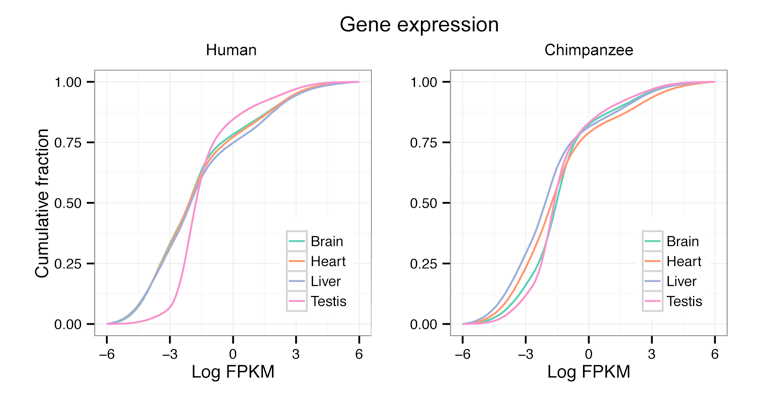

Supplement: S7 Fig — Log10 cumulative density of expression values in assembled genes. Expression is measured in fragments per kilobase per million mapped reads (FPKM) values, selecting the maximum value across all samples. Testis does not show a lack of highly expressed transcripts (actually the opposite is observed for human) that could explain why we detect so many transcripts being expressed in this tissue. (PNG) [file pgen.1005721.s007.png]

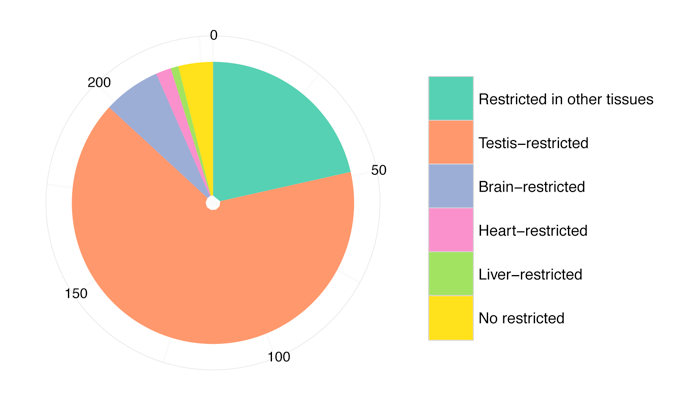

Supplement: S8 Fig — Data is for annotated transcripts in the GTEx catalog which are preferentially expressed in one tissue (tissue-restricted), as measured by a tissue preferential expression index higher than 0.85 (see Methods online for more details on this index). (PNG) [file pgen.1005721.s008.png]

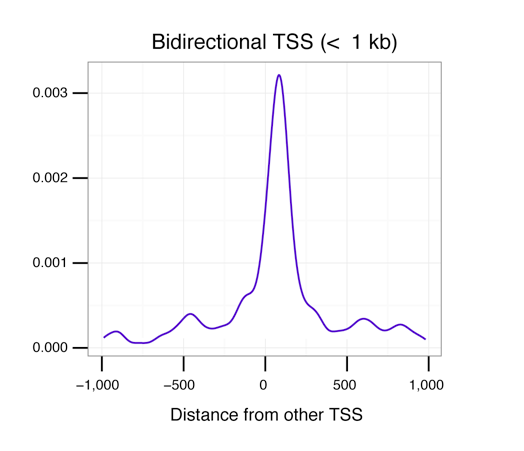

Supplement: S9 Fig — These were defined as antisense genes with the TSSs separated by less than 1 kb, potentially sharing a bidirectional promoter. Negative values imply overlap between the transcripts. There is a strong peak at around 100 nucleotides. (PNG) [file pgen.1005721.s009.png]

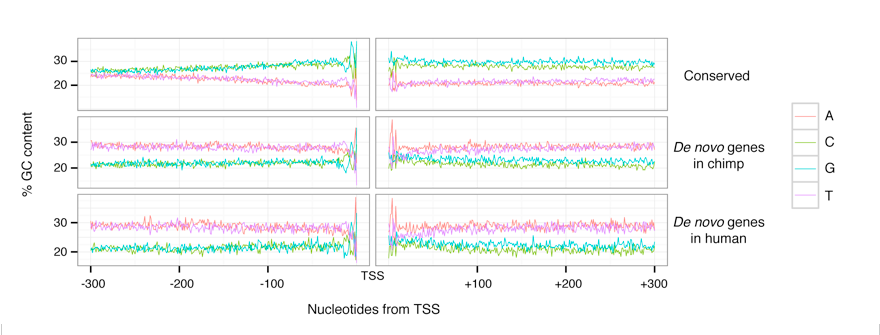

Supplement: S10 Fig — Nucleotide frequencies 300 bp upstream and 300 downstream of the transcription start site (TSS) were calculated for different sets of transcripts. Conserved: 4,323 randomly taken human and chimpanzee annotated transcripts not classified as de novo. (PNG) [file pgen.1005721.s010.png]

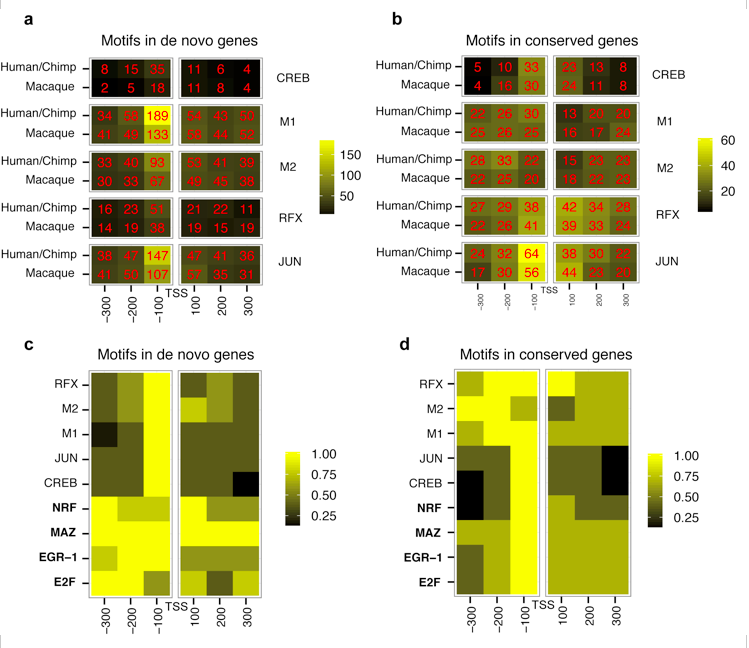

Supplement: S11 Fig — a) Number of matches of overrepresented motifs in 100 bp windows in de novo genes and in the corresponding macaque syntenic regions (corresponds to Fig 3a in main manuscript file). b) Same data for conserved annotated genes. c) Relative motif frequencies in de novo genes including motifs overrepresented in conserved annotated genes in general but not in de novo genes (NRF, MAZ, EGR-1, E2F). d) Data for the same motifs for conserved annotated genes. (PNG) [file pgen.1005721.s011.png]

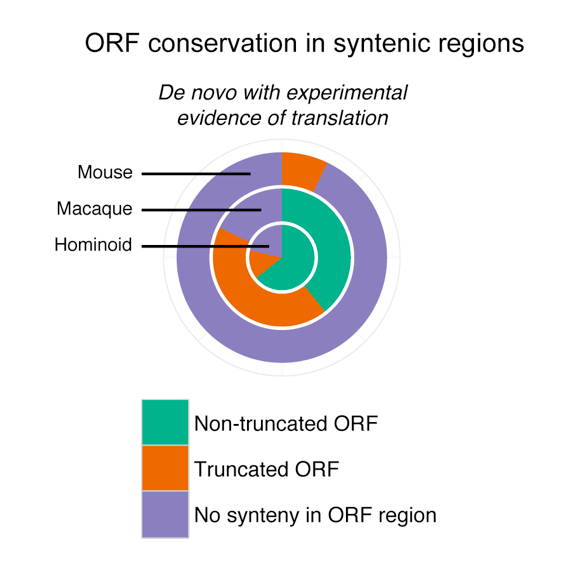

Supplement: S12 Fig — The existence of full or partial synteny was assessed using pairwise genomic alignments from UCSC. Hominoid (inner circle) refers to human when chimpanzee is the reference species and to chimpanzee when human is the reference species. Only ORFs in de novo genes with evidences of preoteogenomics or ribosome profiling are displayed. Non-truncated ORFs are the ones in which the frame, the start codon and the stop codon are conserved in the other syntenic genomic region; otherwise the ORF is truncated. (PNG) [file pgen.1005721.s012.png]
