# Supplementary material for: Origins of De Novo Genes in Human and Chimpanzee
Source: PLoS Genet. 2015 Dec 31;11(12):e1005721. doi: 10.1371/journal.pgen.1005721 (PMC4697840; doi:10.1371/journal.pgen.1005721)
Supplement: S1 Table — Identification of annotated genes in the set of de novo genes was based on the comparison of the genomic coordinates of the assembled transcripts and the genomic coordinates of annotated genes using Cuffcompare. All these genes were hominoid-specific (expressed both in human and chimpanzee). (*) refers to the same orthologous gene in human and chimpanzee. Note that all human coding genes had been annotated as different classes of long non-coding RNAs (lncRNAs) in Ensembl v. 77. (DOC) [file pgen.1005721.s013.doc]

| **Ensembl gene ID** | **Assembly gene ID** | **Number of transcripts** | **Tissue-specificity** | **ORF length (aa)** | **Class** |
| --- | --- | --- | --- | --- | --- |
| ENSG00000224186 | XLOC_155668 | 2 | Testis | 96, 52 | Overlapping antisense |
| ENSG00000253976 | XLOC_193538 | 3 | Testis | 74, 49, 48 | Overlapping Intronic |
| ENSG00000249016 (*) | XLOC_159345 | 1 | Testis | 34 | Intergenic |
| ENSG00000263417 | XLOC_088783 | 3 | Testis | 148, 136, 61 | Intergenic |
| ENSPTRG00000041026 | XLOC_236355 | 1 | Testis | 83 | Intergenic |
| ENSPTRG00000041735 | XLOC_047766 | 1 | Brain | 74 | Overlapping Intronic |
| ENSPTRG00000041069 (*) | XLOC_227215 | 2 | Testis | 34, 34 | Intergenic |
| ENSPTRG00000040082 | XLOC_160846 | 1 | Brain | 35 | Intergenic |
